# Exploring the application of metal-based photothermal agents in photothermal therapy combined with immune checkpoint therapy

**DOI:** 10.3389/fphar.2025.1553158

**Published:** 2025-02-13

**Authors:** Bin Xie, ZhiRong Xiao, JiaBao Ling, Yichao Peng, Tianfeng Chen

**Affiliations:** Department of Pharmacy and General Surgery of Puning People’s Hospital (Guangdong Postdoctoral Innovation Practice Base of Jinan University), College of Chemistry and Materials Science, MOE Key Laboratory of Tumor Molecular Biology, Jinan University, Guangdong, China

**Keywords:** noble metal, 2D transition metal dichalcogenides, photothermal therapy, immune checkpoint blockades, immune checkpoint therapy

## Abstract

Photothermal therapy (PTT), a popular local treatment that uses heat to ablate tumors, has limited efficacy in addressing metastatic and deeply located tumors when used alone. Integrating PTT with immunotherapy not only yields a synergistic effect but also promotes cancer regression and confers the benefit of immune memory, which can surmount the challenges faced by PTT when used in isolation. Metal-based nanomaterials, renowned for their superior photothermal conversion efficiency and distinctive photochemical properties, have been extensively researched and applied in the field of PTT. This review summarizes the latest developments in combination therapies, with a specific focus on the combination of PTT and immune checkpoint therapy (ICT) for cancer treatment, including a comprehensive overview of the recent advancements in noble metal-based and 2D transition metal chalcogenides (TMDCs)-based photothermal agents, and their anticancer effect when combining PTT with immune checkpoint blockades (anti-CTLA-4 and anti-PD-L1) therapy. The goal of this review is to present an overview of the application, current challenges and future prospects of metal-based photothermal agents in PTT combined with ICT for cancer treatment.

## 1 Introduction

Cancer is one of the most leading causes of death worldwide, in 2022, nearly 20 million new cancer cases were diagnosed, and approximately 10 million people died from the disease (Bray et al., 2024). It is projected that about one in five individuals will develop cancer, with one in nine men and one in twelve women succumbing to it. The primary therapeutic approaches for cancer management include surgery, chemotherapy, radiotherapy, and other methods (Debela et al., 2021; Liu B et al., 2024). However, these traditional treatments have several limitations and can cause significant side effects, which make it necessary to explore more safety and effective treatment strategies for cancer treatment.

Photothermal therapy (PTT) is a local treatment that uses photothermal agents (PTAs) to harvest energy from near-infrared (NIR) light and convert the energy into heat, which leads to an increase in the temperature surrounding the tumor tissue ultimately inducing the death of cancer cells (Yang et al., 2016; Liu et al., 2018; Nam et al., 2018; Gao et al., 2022). At present, the photothermal agents mainly include noble metal nanomaterials (Wang et al., 2012; Ding et al., 2021), graphene (Lin et al., 2020; Li L et al., 2024), two-dimensional transition metal chalcogenides (TMDCs) (Gong et al., 2017), semiconducting polymer nanoparticles (Zhang Y et al., 2018), organic small molecule dyes and so on, some of these summarized in Table 1. Among these photothermal agents, noble metal-based and TMDCs-based photothermal agents possess numerous advantageous characteristics, including high photothermal conversion efficiencies and unique electronic and optical properties (Hernández and Galarreta, 2019; Zhang X. et al., 2019). Moreover, their large surface area and low cytotoxicity facilitate the entry of biomolecules and drugs into cells, which is crucial for effective cancer therapy (Gong et al., 2017; Yang F et al., 2024). This treatment modality has a high degree of controllability in the treatment of tumors, and can also produce a large number of relevant antigens when eliminating the primary tumor. At the same time, as a non-invasive therapeutic method, PTT has the advantages of high specificity, low side effects, and high photothermal conversion efficiency, exhibiting a good ablation effect on solid tumors (Ban et al., 2017; Zhang et al., 2021; Kong and Chen, 2022). Therefore, it is widely favored by scientific researchers.

**TABLE 1:** The types of metal-based photothermal agents that have been reported.

Photothermal agents	Main elements	Wavelength (nm)	Power (W/cm^2^)	Cancer cell type	References
Cu@Cu_2_O@polymer NPs	Cu	660	0.61	HeLa	Tai et al. (2018)
MoS_2_-PEG	Mo, S	808	1.0	4T1	Feng et al. (2015)
BSA-Cu_2_SeNPs-DOX	Cu, Se	808	3.0	U251	Liu et al. (2018)
SnSe-PVP nanorods	Sn, Se	808/1,064	1.0	4T1	Tang Z et al. (2018)
Cu_2_MoS_4_ NPs	Cu, Mo, S	808	0.48	U14	Chang et al. (2019)
MoSe_2_	Mo, Se	808	2.0	A375	He et al. (2019)
WS_2_-PEG	W, S	808	0.5	HeLa	Kong et al. (2019)
Bi-MoSe_2_@PEG-Dox	Bi, Mo, Se	808	2.0	HepG2	Wang et al. (2019)
Au@Se NPs	Au, Se	808	1.0	U14	Wang et al. (2020)
Ti_2_N QDs	Ti, N	808/1,064	1.0	4T1/U87	Shao et al. (2020)
NbS_2_-PVP	Nb, S	808/1,064	1.5	4T1	Sun Y et al. (2021)
PBPTV@mPEG (CO)	Fe	808	2.5	4T1	Ma et al. (2022)
Te-PEG NSs	Te	808	2.0	4T1	Pan et al. (2022)
RRP-MPBA-GNRs	Au	808	2.0	A549, HepG2	Lin et al. (2024)
PyAnOH-Ag	Ag	840	1.5	HepG2	Huang et al. (2019)
MoO_3-x_ NWs	Mo, O	980	1.0	HepG2	He et al. (2024)
Pt-Mn-PEI	Pt, Mn	980	0.5, 0.7	4T1	Li et al. (2024)
Mo_2_C	Mo, C	1,060	1.0	HeLa	Liu et al. (2019)
Zn_4_-H_2_Pc/DP NPs	Zn	1,064	0.6	MCF-7	Pan et al. (2019)
AuNRs@SiO_2_-RB@MnO_2_	Au, Si, Mn	1,064	1.0	4T1	Wen et al. (2022)

However, due to the limited penetration of light in tissues and inefficacy target ability to tumor site, the use of photothermal therapy alone will face problems such as low thermal ablation efficiency of primary tumors in deep tissues and unsatisfactory treatment effect for diffuse metastatic tumors (Chu and Dupuy, 2014; Deng et al., 2021). Therefore, scientists are working on strategies to combine photothermal therapy with other treatments. At present, the combination of PTT with photodynamic therapy (PDT), chemotherapy and immunotherapy are widely studied (Li et al., 2020; Huang et al., 2021; Liu et al., 2022; Li S et al., 2024). The absorption spectra of photodynamic and photothermal agents often do not align, which requires the use of two different lasers for long-term combination treatments involving PTT and PDT, further complicating the treatment process (Yang et al., 2017; Younis et al., 2019). The combination of PTT with immunotherapy effectively addresses the aforementioned issues while also overcoming the challenges of possible resistance to chemotherapy drugs. Therefore, researchers are committed to studying the combination of photothermal therapy and immunotherapy.

In recent years, immunotherapy has emerged as a promising cancer treatment option for individuals seeking to leverage their own immune system against diseases, particularly for those with uncontrollable cancers (Tan et al., 2020; Zhang and Zhang, 2020; Li et al., 2023; Liu Y et al., 2024). Tumors utilize multiple strategies to avoid immune detection, including the activation of regulatory T cells and the induction of inhibitory checkpoint receptors (Gajewski et al., 2013). Immune checkpoint proteins, including cytotoxic T lymphocyte-associated molecule-4 (CTLA-4) and programmed cell death ligand-1 (PD-L1) which are able to cause T cells with tumor-killing inactivation and promotion of cancer cell growth and invasion. Thus, the immune checkpoint blockades (ICBs), anti-CTLA-4 and anti-PD-L1, have been employed to suppress malignant tumors and have significantly improved patient survival rates (Doroshow et al., 2021; Matias et al., 2021). However, the eradication of the primary tumor through single immunotherapy remains a significant challenge, especially due to the considerable antigenic variation observed across different cancers (Copier and Dalgleish, 2006; Shao et al., 2015). In addition, PTT not only generates endogenous tumor-associated antigens (TAAs) during thermal ablation of primary tumors, but also generates damage-associated molecular patterns (DAMPs) and induces immunogenic cell death (ICD) in tumors. TAAs enable personalized and specific immunotherapy, and ICDs can awaken the immune system and enhance the immunogenicity of tumors (Guo et al., 2022; Zhuang et al., 2023). Therefore, the combination of immunotherapy with PTT is the most promising strategy to overcome the limitations of monotherapy (Wu et al., 2022b). In this review, we will summarize the recent application in the synergistic treatment of cancer with mental-based PTT and immune checkpoint therapy (ICT). We hope this review can provide a comprehensive status of mental-based PTT combined ICT for further development, and contribute to the early clinical translation of metal-based photothermal agents.

## 2 Immune checkpoint blockades (ICBs)

**FIGURE 1:**
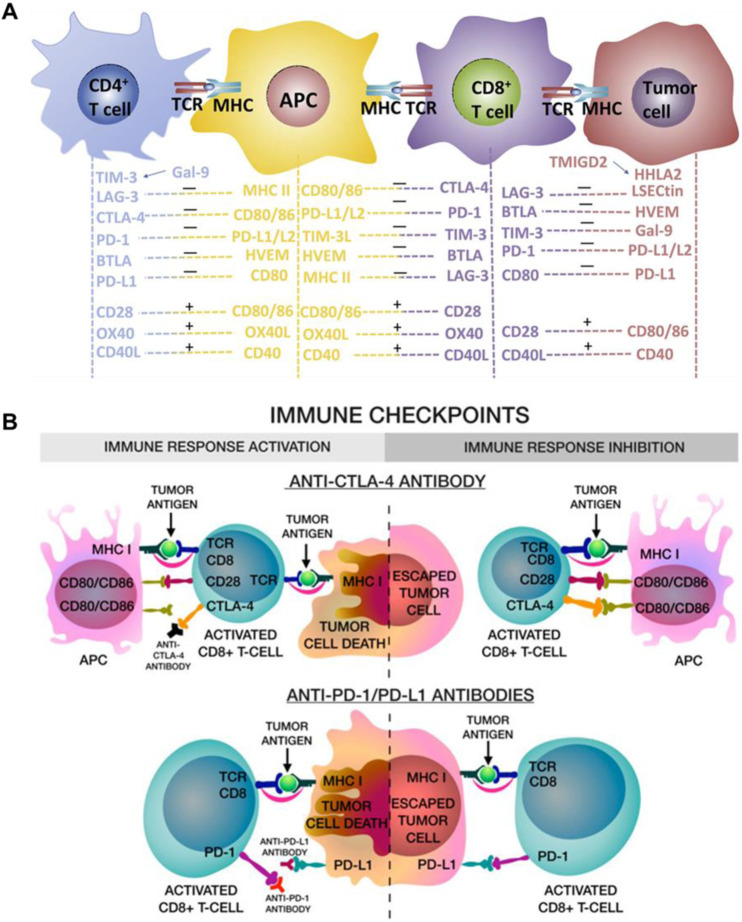
Immune checkpoints blockade (anti-CTLA-4 and anti-PD-L1) enhanced the immune cells activation and eradicate tumor cells. **(A)** The schematic illustration of the multi-signal processes of T-cell activation and inactivation. Reproduced with permission from Ref (Jiang et al., 2021). Copyright 2019 Chongqing Medical University. Production and hosting by Elsevier B.V. **(B)** The schematic illustration of anti-CTLA-4 blocks the binding of CD80/CD86 in APC cells, thus activated CD8⁺ CTLs can inhibit tumor cells proliferation, and the anti-PD-L1 blocks the binding, leading to tumor cells be killed by CD8^+^ T cells. Reproduced with permission from Ref (Filin et al., 2020). Copyright 2020 by the authors.

Over the past decades, a multitude of immune checkpoint proteins have been identified and studied, including programmed cell death protein-1 (PD-1)/PD-L1, CTLA-4, lymphocyte-activation gene 3 (LAG3), T cell immunoglobulin and mucin domain containing-3 (TIM3), T cell immunoreceptor with Ig and ITIM domains (TIGIT), and B- and T-lymphocyte attenuator (BTLA) (He and Xu, 2020). Normally, these immune checkpoints could protect normal cells from damage through activating the immune system to resist the malignancies and infections (Marin-Acevedo et al., 2018). However, in tumor cells, PD-1/PD-L1 and CTLA-4, the most broadly studied immune checkpoints, lead to T cells with tumor-killing inactivation and promote tumor cells growth and invasion (Figure 1A) (Jiang et al., 2021; Liu et al., 2021; Song et al., 2021; Sun S et al., 2021; Yang et al., 2024). As shown in Figure 1B, CTLA-4 combines with CD80 and CD86 ligands on dendritic cells (DCs) to deliver an inhibitory signal, which regulates the expression of Treg cells, an immunosuppressive T cell, and inhibits T cell immune response activation. Besides, the PD-L1 in cancer cells combines with PD-1, expressed on T cells, prevents the T cells from killing cancer cells, causing the immune escape of tumor cells (Filin et al., 2020). Therefore, the immune checkpoint blockades (ICBs), anti-CTLA-4 and anti-PD-L1, can enhance antitumor immunity through disrupting the tumor co-inhibitory immune cells. Although ICT is an effective strategy to activating T cells and promoting anti-tumor immune responses, if the number of immune cells is low or non-existent in the tumor microenvironment, it will diminish the therapeutic effect of ICBs (He and Xu, 2020). Photothermal therapy (PTT), using photothermal agents to generate heat under laser irradiation, can induce ICD in tumors to promote T cells infiltration. Therefore, combining PTT with ICB treatment can enhance immune cells infiltration in tumor and prevent tumor metastases. A comprehensive summary of the noble metal-based and 2D TMDCs-based photothermal agents in PTT combined with ICT are showed in Table 2.

**TABLE 2:** The summary of noble metal-based and TMDCs-based photothermal agents in PTT combined with ICT.

Therapy types	Photothermal agents	Immune checkpoint	Effector cells	Power and time	Temperature	References
PTT/ICT	Au@Pt-LM^D^P	PD-L1	CD8^+^ T cells, CD4^+^ T, Tregs	1.5 W/cm^2^, 5 min	56.5°C	Yang et al. (2019)
PTT/ICT	AuNDs@aPD-1	PD-L1	CD8^+^ T cells, CD4^+^ T, effector memory T cells, Tregs	2.0 W/cm^2^, 10 min	59.3°C	He et al. (2022)
PTT/PDT/ICT	Au/Ag NRs	PD-L1, CTLA-4	CD8^+^ T cells, effector memory T, Tregs	1.0 W/cm^2^, 10 min	Approx. 55°C	Jin et al. (2021)
PTT/ICT	AgPP@P@M	PD-L1	CD8^+^ T cells, CD4^+^ T cells	0.5 W/cm^2^, 6 min	49.44°C ± 0.45°C	Xiong et al. (2024)
PTT/ICT	AuPtAg-PEG-GOx	PD-L1	CD8^+^ T cells, CD4^+^ T cells, Tregs, macrophages	0.7 W/cm^2^, 5 min	43.0°C	Wang et al. (2022)
PTT/ICT/CT	Pd-Dox@TGMs	PD-L1	CD8^+^ T cells, Tregs	0.5 W/cm^2^, 5 min	51.2°C	Wen et al. (2019)
PTT/ICT	RBC-MoSe_2_	PD-L1/PD1	CD8^+^ T cells, CD4^+^ T cells, macrophages	2.0 W/cm^2^, 3 min	53.5°C	He et al. (2019)
PTT/ICT	MoSe_2_-DPEG	PD-L1	CD8^+^ T cells, CD4^+^ T cells, Tregs	1.0 W/cm^2^, 5 min	45°C	Huang Z et al. (2024)
PTT/ICT/CT	FPMF@CpG ODN	CTLA-4	CD8^+^ T cells, CD4^+^ T cells, effector memory T cells, Tregs	2.0 W/cm^2^, 5 min	54.6°C	Zhang X. et al. (2019)
PTT/ICT/CT	1-MT-Pt-PPDA@MoS_2_	PD-L1	CD8^+^ T cells, CD4^+^ T cells, Tregs	1.0 W/cm^2^, 5 min	61°C	Hu et al. (2021)
PTT/ICT/RT	WO_2.9_-WSe_2_-PEG	PD-L1	CD8^+^ T cells, CD4^+^ T cells	1.0 W/cm^2^, 8 min	Approx. 48°C	Dong et al. (2020)

## 3 Noble metal-based photothermal agents

Noble metals, primarily consisting of gold, silver, platinum and palladium, not only possess synthetic tunability and strong localized surface plasmon resonance (LSPR) effects (Jahangiri-Manesh et al., 2022; Yu et al., 2024) but also have superior optical and photothermal properties. Based on the aforementioned outstanding properties of noble metal, noble metal-based nanomaterials are widely used in PPT for cancer treatment.

### 3.1 Gold-based nanomaterials

**FIGURE 2:**
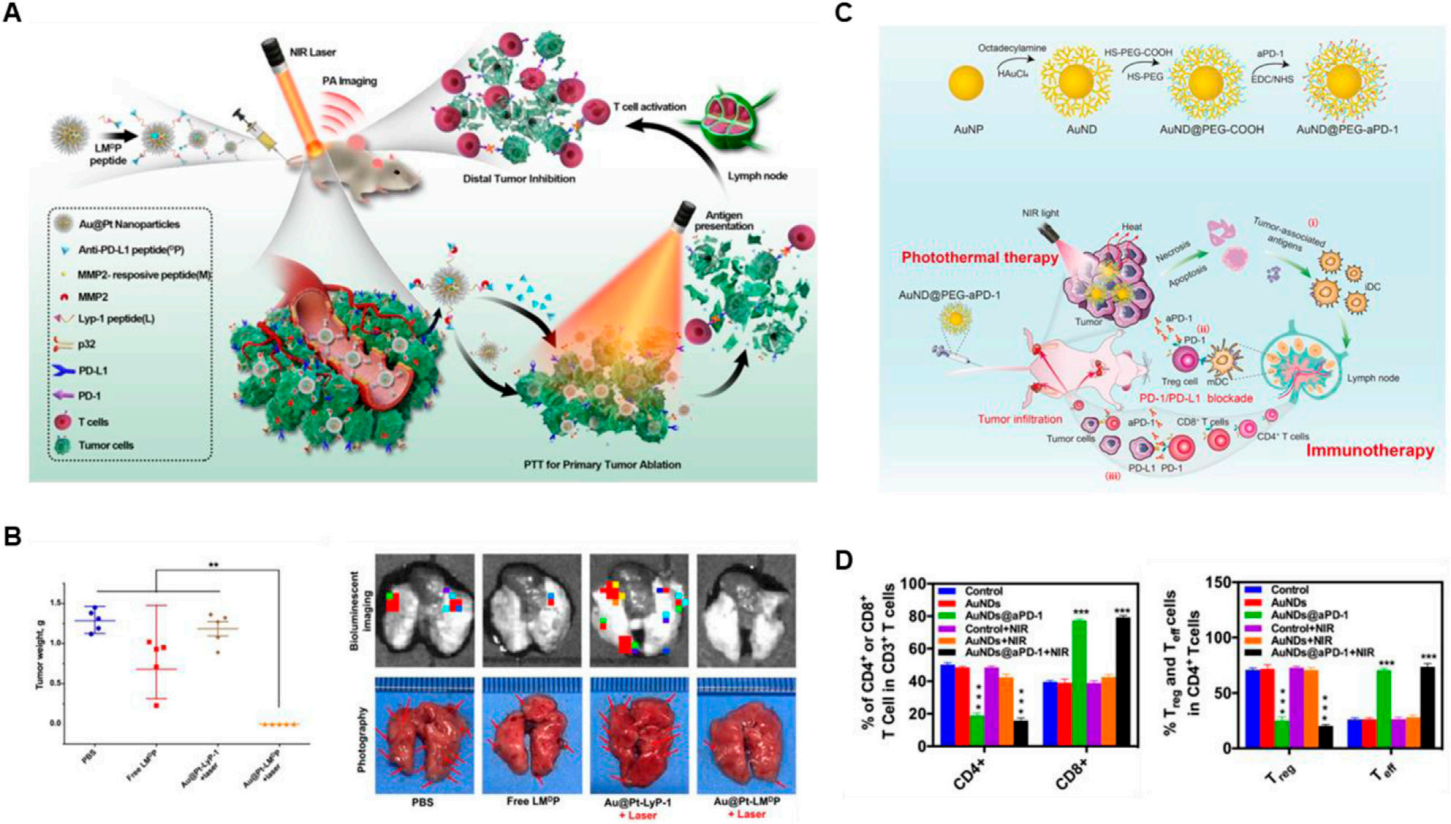
Gold-based nanomaterials combined with PTT and ICBs enhanced the anticancer ability. **(A)** The schematic illustration of Au@Pt-LM^D^P combined with photothermal and immunotherapy. **(B)** The weights of tumors and the bioluminescent imaging of the metastatic foci of 4T1 lung metastatic tumors at the end of treatments. Reproduced with permission from Ref (Yang et al., 2019). Copyright 2019, Elsevier B.V. All rights reserved. **(C)** The schematic illustration of AuNDs@aPD-1 in photothermal therapy combined with immune checkpoint therapy *in vivo*. **(D)** The quantitative analysis of CD4^+^ T cells, CD8^+^ T cells, Treg cells and Teff cells in tumor at the end of treatments were detected by Flow cytometry. Reproduced with permission from Ref (He et al., 2022). 2022, Elsevier Ltd. All rights reserved.

Gold nanomaterials are the most widely studied noble metal photothermal agents, due to their outstanding photostability, high biocompatibility, and considerable photothermal conversion efficiency (Gui et al., 2021; Shuai et al., 2023). In order to endow gold-based nanomaterials with targeting and controlled drug release, Yang et al. combined Au@Pt nanoparticles with the antagonist LM^D^P peptide to form a multifunctional nanosystem (Au@Pt-LM^D^P) (Yang et al., 2019). As shown in Figures 2A, B, the LM^D^P peptide consists of a three-part combination including a PD-L1 antagonist short D-peptide (^D^PPA-1), a MMP2-reactive peptide (PLGVRG) and LyP-1 (CGNKRTRGC) sequence identical to tumor homing ligand, which endow Au@Pt-LM^D^P with high tumor inhibition and the alleviation of metastasis, especially the lung metastasis, via enhancing population of the CD8^+^ T cells and downregulating the Treg activity when combination with PTT. Besides, He et al*.* prepared AuNDs@aPD-1, which not only exhibits long-term retention of drugs and enhancing the tumor-targeting ability, but also efficiently promotes the activation of antitumor CD4^+^ and CD8^+^ T cells, and inhibit the Treg cells activity in tumor when combined with PTT, showing significant anticancer ability to local as well as distant tumors (Figures 2C, D) (He et al., 2022).

### 3.2 Silver-based nanomaterials

**FIGURE 3:**
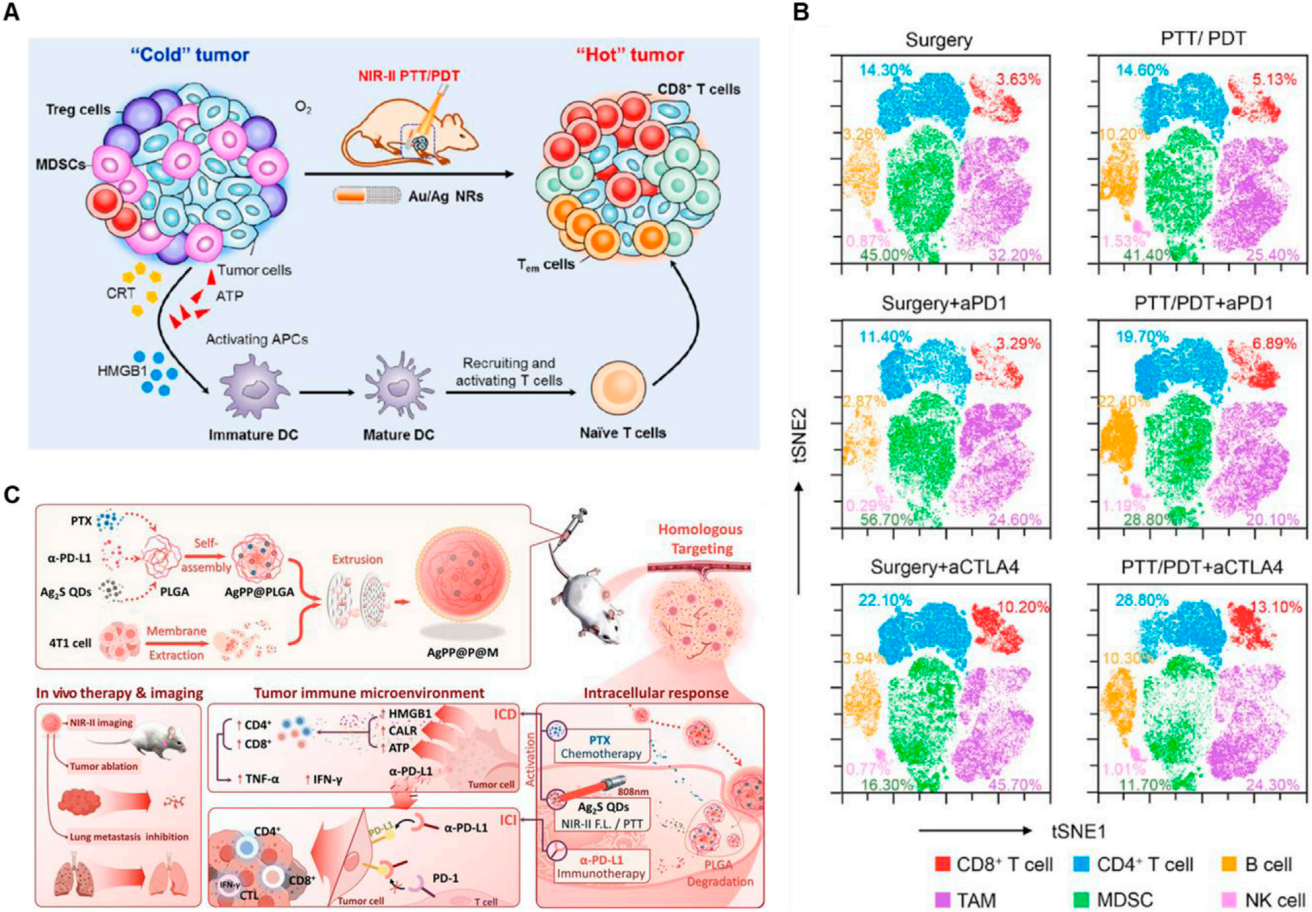
Silver-based nanomaterials combined with PTT and ICBs enhanced the anticancer ability. **(A)** Schematic illustration of anticancer immune responses induced by Au/Ag NR combined with NIR-II PTT/PDT. **(B)** t-SNE analysis of immune cells in tumors at the end of treatment. Reproduced with permission from Ref (Jin et al., 2021). Copyright 2020, Elsevier Ltd. All rights reserved. **(C)** Schematic illustration of AgPP@P@M NPs enhanced immunotherapy *in vivo*. Reproduced with permission from Ref (Xiong et al., 2024). Copyright 2024, Wiley-VCH GmbH.

Silver nanomaterials have been used in the field of PTT for their low toxicity, easy preparation, tunable SPR band, and superior thermal conductivity compared to other metals (Boca et al., 2011). Jin et al*.* found that a corn-like Au/Ag nanorod (NR) combination with 1,064 nm laser irradiation obviously increased the tumor infiltration of T cells after the injection of anti-PD-1 or anti-CTLA4, indicating a strong immunological memory effect and high anticancer ability (Figures 3A, B) (Jin et al., 2021). Moreover, in order to effective target the tumor site and realize the controllable TME-responsive drug release, Xiong et al*.* constructed Ag_2_S QDs/PTX/α-PD-L1@PLGA@membrane (AgPP@P@M) NPs, which exhibited superior antitumor ability and lung metastases inhibition for primary TNBC by enhancing ICD and activating the activity of CD4^+^ and CD8^+^ T cells, suggesting excellent synergistic therapeutic effects combined PTT with ICB (Figure 3C) (Xiong et al., 2024).

### 3.3 Platinum-based nanomaterials

**FIGURE 4:**
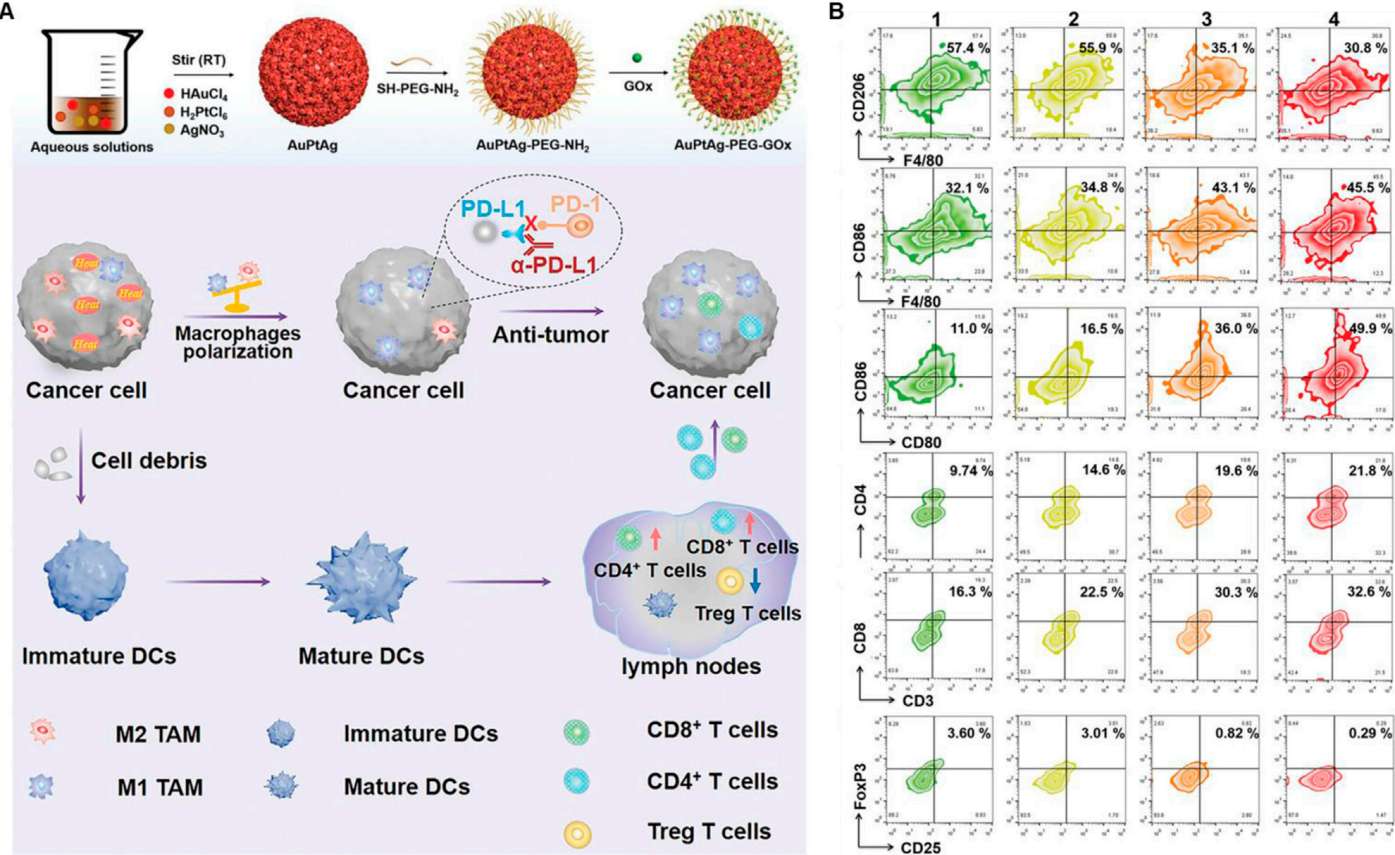
Platinum-based nanomaterials combined with PTT and ICBs enhanced the anticancer ability. **(A)** Schematic illustration of the AuPtAg-PEG-GOx nanozyme combined with 1,064 nm laser irradiation and anti-PD-L1 *in vivo*. **(B)** Flow cytometric analyses of the populations of M2 and M1 macrophages in tumor, DC cells in lymph nodes as well as CD4^+^ T cells, CD8^+^ T cells, and Treg in spleen. Reproduced with permission from Ref (Wang et al., 2022). Copyright 2022, The Authors. Advanced Science published by Wiley-VCH GmbH.

Platinum nanoparticles with properties including DNA damage (Manikandan et al., 2013), antioxidant activity (Wang et al., 2023) and light absorption in the biological range (Depciuch et al., 2020) show high potential for use as effective photosensitizers in PTT. In addition, platinum nanoparticles can also be used in combination with photoacoustic imaging, magnetic resonance imaging and immunotherapy due to its excellent photo and thermal stability (Zhao et al., 2017). As demonstrated in Figures 4A, B, Wang et al. constructed AuPtAg-PEG-GOx nanozyme by a one-step method, which showed a broad absorption band in the near-infrared (NIR) region and higher CAT-like activity (Wang et al., 2022). More importantly, AuPtAg-PEG-GOx can effectively reprogram “cold” tumors into “hot” tumors through enhancing the expression of M1 macrophages in tumors after combination with 1,064 nm laser irradiation and anti-PD-L1, and increasing the ratio of DC maturation in lymph nodes, CD4^+^ and CD8^+^ T cells activation in spleen as well as inhibiting the proportion of Treg cells, showing high tumors inhibition.

### 3.4 Palladium-based nanomaterials

**FIGURE 5:**
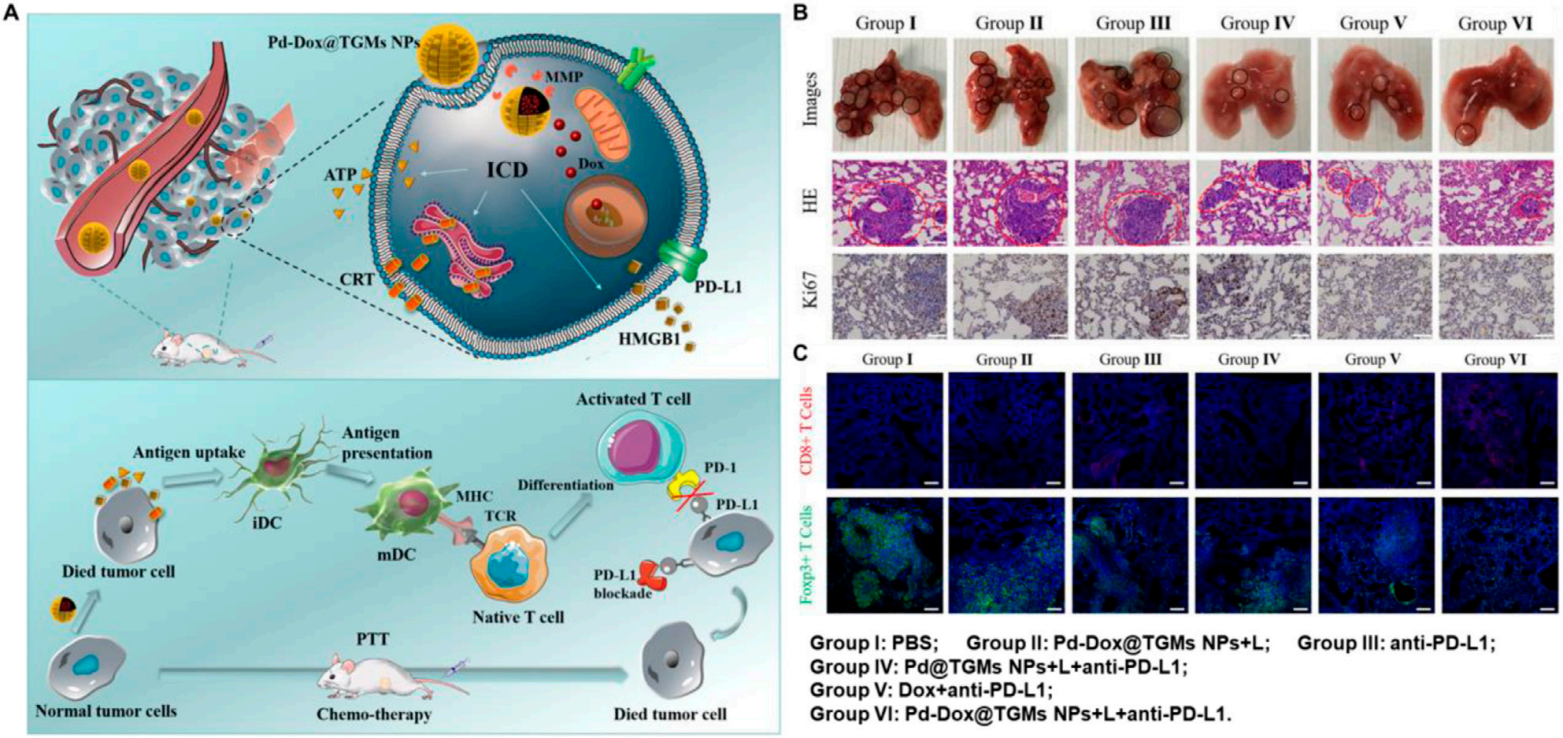
Palladium-based nanomaterials combined with PTT and anti-PD-L1 enhanced the anticancer ability. **(A)** Schematic illustration of the Pd-Dox@TGMs NPs nanozyme combined with 808 nm laser irradiation and anti-PD-L1 *in vivo*. **(B)** The pictures of lungs and staining of H&E and Ki67 in lung sections at the end of treatment. **(C)** The infiltration of CD8^+^ T cells and Tregs in lung sections after treatment. Reproduced with permission from Ref (Wen et al., 2019). Copyright 2019, American Chemical Society.

Palladium-based nanomaterials have been used in chemotherapy (CT), photodynamic therapy (PDT) and PTT due to its high catalytic activity in the presence of hydrogen peroxide, which facilitates the formation of oxygen molecules, thereby enhancing the efficiency of various cancer treatment (Wei et al., 2018; Zhang J et al., 2018; Singh et al., 2023; Yin et al., 2023; Lakkakula et al., 2024). Moreover, it has been demonstrated that palladium-based nanomaterials used in synergy with PPT and ICT can effectively activate the immune response to suppress tumor growth and metastasis. For instance, Wen et al. constructed Pd-Dox@TGMs NPs with an excellent inhibitory effect on CT26 tumor cells due to the synergistic therapy of CT and PTT, which cause ICD in CT26 cells (Figure 5A) (Wen et al., 2019). In addition, as shown in Figures 5B, C, the nanosystem could effectively trigger CD8^+^ T cells and suppress the tumor growth in the CT26 lung metastatic model when combining with anti-PD-L1. The Pd-Dox@TGMs NPs demonstrate remarkable antitumor properties of palladium-based photothermal agents when combining with PTT and ICT, however, there is still a dearth of research in this area for other palladium-based photothermal agents.

In conclusion, although the gold, silver, platinum and palladium-based metal photothermal agents have exhibited effectively anticancer ability *in vitro* and *in vivo* when combined PTT with ICT, high price, poor targeting specificity to tumor tissue, complex synergistic therapy processes and other shortcomings remain to be overcome.

## 4 Two-dimensional transition metal dichalcogenides

**FIGURE 6:**
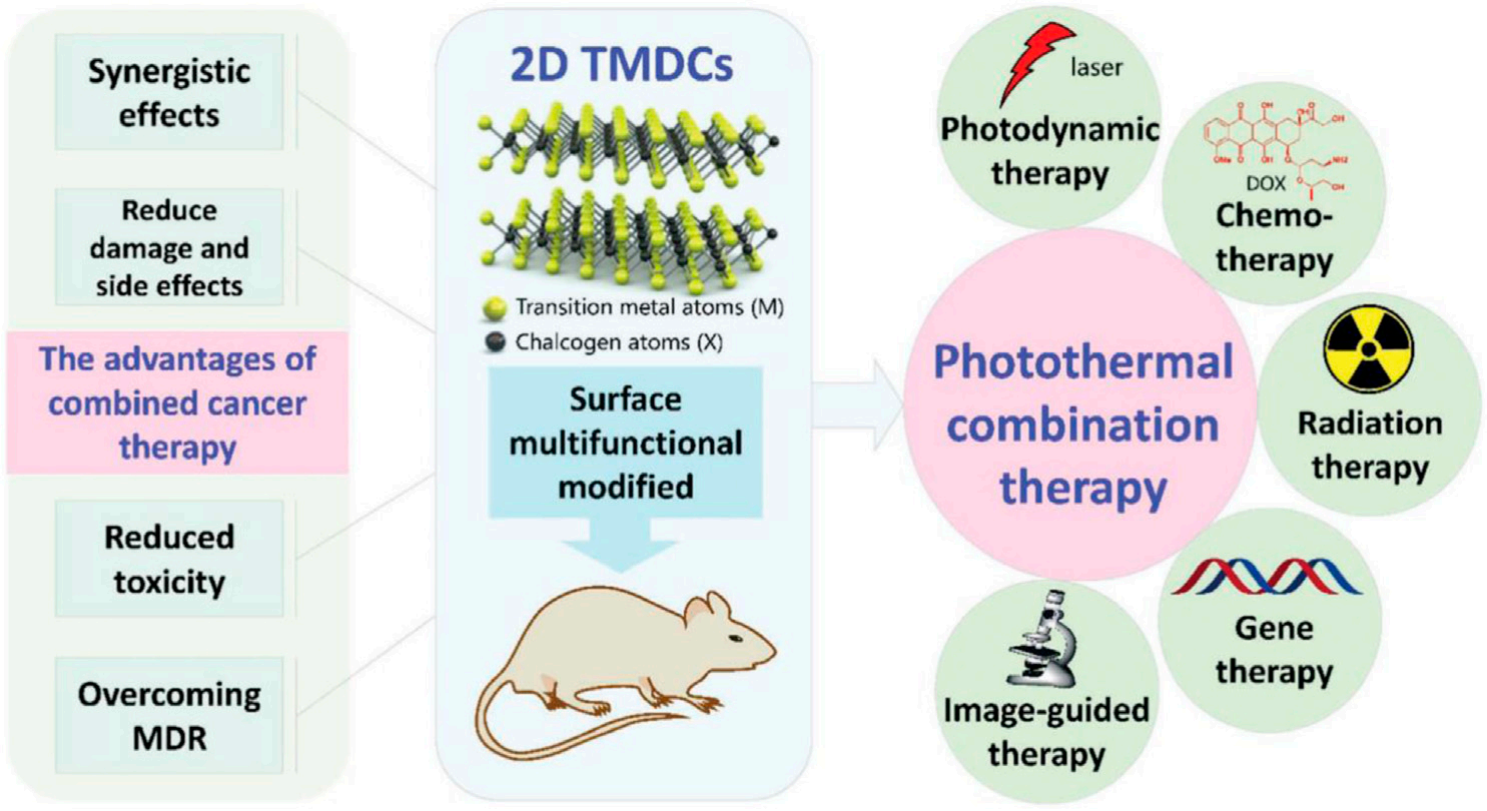
The advantages of 2D TMDCs combined with photothermal therapy. Reproduced with permission from Ref (Gong et al., 2017). Copyright 2017, Royal Society of Chemistry All rights reserved.

Two-dimensional nanomaterials exhibit high NIR absorption ability, photothermal conversion efficiency and surface area, which hold huge potential for drugs delivery and photothermal tumor treatment (Wu et al., 2022a). Among many two-dimensional nanomaterials, the extremely high surface area of two-dimensional transition metal disulfide compounds (2D TMDCs) is able to attach a variety of nanoparticles and fluorescent probes for integration with other functional moieties, giving them richer properties than other 2D nanomaterials. The structure of TMDCs is mainly composed of two chalcogen atoms X (such as S, Se, Te) and a transition metal atom M (such as V, Ti, Nb, Mo, Zr, Co., Hf, Mn, Ta, etc.) to form a sandwich-like X-M-X (MX_2_) structure (Gong et al., 2017; Jiang et al., 2022; Mondal and De, 2024). In recent years, the combined anticancer advantage of 2D TMDCs have also gained more attention in the area of biomedicine because of its excellent properties with low toxicity, improving therapeutic efficacy and overcoming multi-drug resistance (MDR), which was shown in Figure 6 (Gong et al., 2017). So far, 2D TMDCs, such as MoS_2_, WS_2_, MoSe_2_ and WSe_2_ have been extensively studied for synergistic therapy, including photothermal therapy/photodynamic therapy (PTT/PDT) (Liu et al., 2014; Jia et al., 2017; Liu et al., 2020; Zhao et al., 2024), photothermal therapy/chemotherapy (PTT/CT) (Long et al., 2020; Gao et al., 2021; Chen et al., 2023; Xie et al., 2023), photothermal therapy/radiation therapy (PTT/RT) (Wang et al., 2016; Qi and Liu, 2019) and photothermal therapy/gene therapy (PTT/GT) (Kim et al., 2016; Zhang et al., 2016). However, there are relatively few studies focusing on PTT combined with ICT using 2D TMDCs as photothermal agents. Therefore, three 2D TMDCs (MoSe_2_, MoS_2_ and WSe_2_), which have demonstrated a high capacity to activate immune cells and realize effective tumor eradication, are introduced as follow.

### 4.1 MoSe_2_-based nanomaterials

**FIGURE 7:**
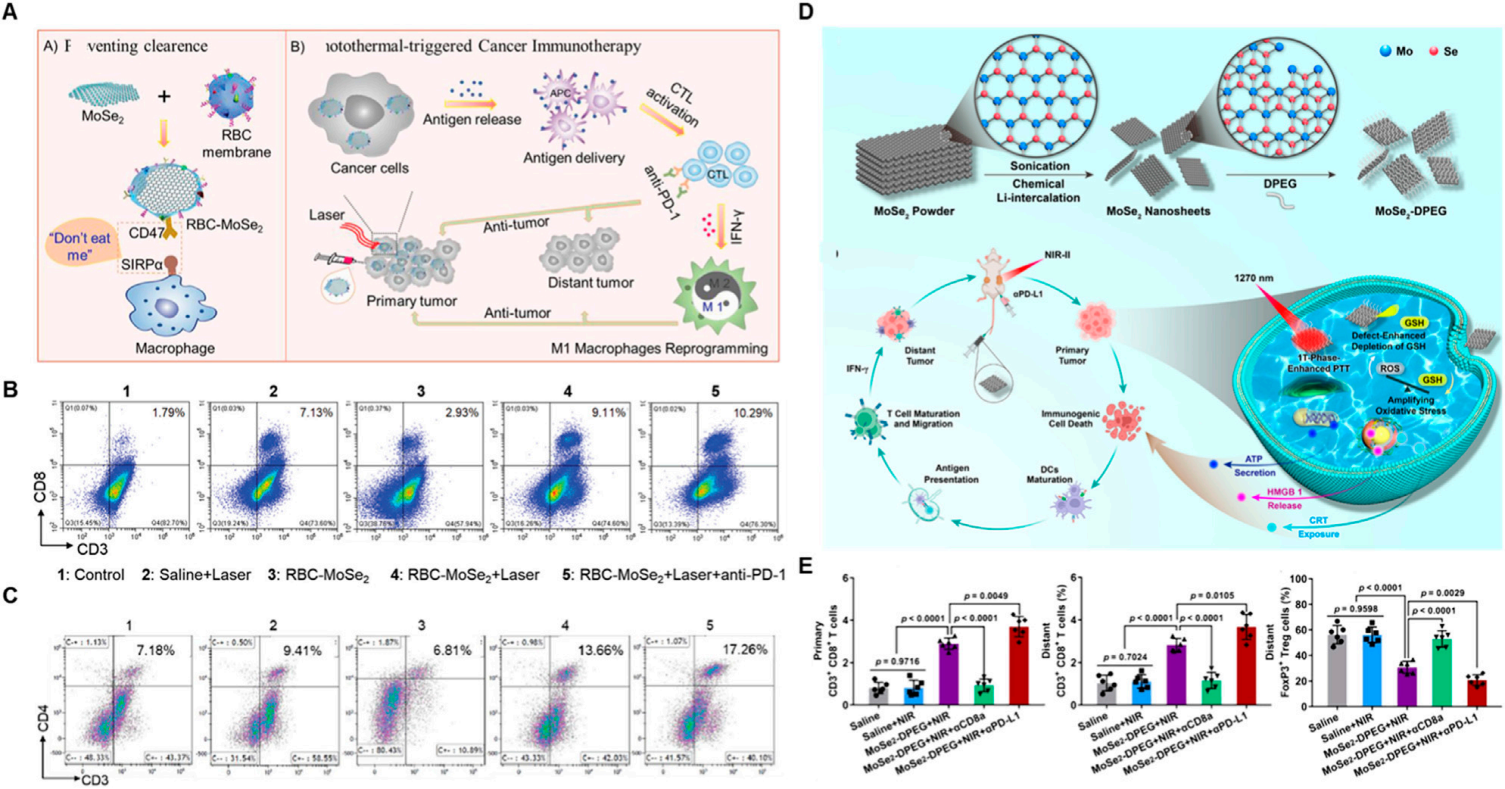
MoSe_2_-based nanomaterials combined with PTT and ICBs enhanced the anticancer ability. **(A)** Schematic illustration of RBC-MoSe_2_ nanosheet combined with 808 nm laser irradiation and anti-PD-L1, which efficiently enhanced photothermal-triggered cancer immunotherapy. Flow cytometric analyses of the CD8^+^T cells in primary tumor **(B)** and the CD4^+^ T cells in spleen **(C)** at the end of different treatment. Reproduced with permission from Ref (He et al., 2019). Copyright 2019, WILEY-VCH Verlag GmbH and Co. KGaA, Weinheim. **(D)** Schematic illustration of MoSe_2_-DPEG combined with 1,270 nm laser irradiation and anti-PD-L1 *in vivo*. **(E)** The quantitative analysis of CD8^+^ T cells in primary and distant tumor, and the Treg cells in distant tumor. Reproduced with permission from Ref (Huang H et al., 2024). Copyright 2024, American Chemical Society.

MoSe_2_ is considered as a promising 2D TMDCs for its narrow band gap (∼1.05 eV), considerable layer spacing (∼0.65 nm), excellent biocompatibility and highly efficient NIR-II absorption (Yuwen et al., 2016; Jiang et al., 2022). He et al. developed two-dimensional MoSe_2_ nanosheets with high photothermal conversion efficiency through employing a liquid exfoliation method (He et al., 2019). As shown in Figures 7A–C, hemocompatibility and circulation time were enhanced by coating two-dimensional MoSe_2_ nanosheets with red blood cell (RBC) membranes, which had excellent photothermal conversion efficiency to prevent macrophage phagocytosis. In addition, the RBC-MoSe_2_ combined with 808 nm laser irradiation and PD-1 checkpoint-blockade exhibited higher tumor ablation through CD4^+^ T cells activation in primary tumor and reprogramming the tumor-associated macrophages to tumoricidal M1 phenotype. Similarly, Huang et al. constructed MoSe_2_-DPEG nanosheets, which performance high PTT synergizing with anti-PD-L1, thus efficient depleting GSH, improving the response of ICB and stimulating CD8^+^ T cell-mediated systemic antitumor immune responses (Figures 7D, E) (Huang Z et al., 2024). However, circulating tumor cells (CTCs), detaching from the primary tumor tissue and entering bloodstream, are vital for the development and progression of lung cancer, based on this, Huang et al. constructed a PD-L1-MFP NS nanosystem, which is based on MoSe_2_ as a core and Fe_3_O_4_ as a functional magnetic material encapsulated with PDA coating in combination of anti-PD-L1 (Huang H et al., 2024). The nanosystem is able to enrich CTCs by an outer magnetic field and killing the captured CTCs under the irradiation of NIR laser. Furthermore, the nanosystem was also able to activate the expression of the surface ligands of NKG2D, enabling NK cells to kill CTCs, further preventing CTCs from undergoing immune escape.

### 4.2 MoS_2_-based nanomaterials

**FIGURE 8:**
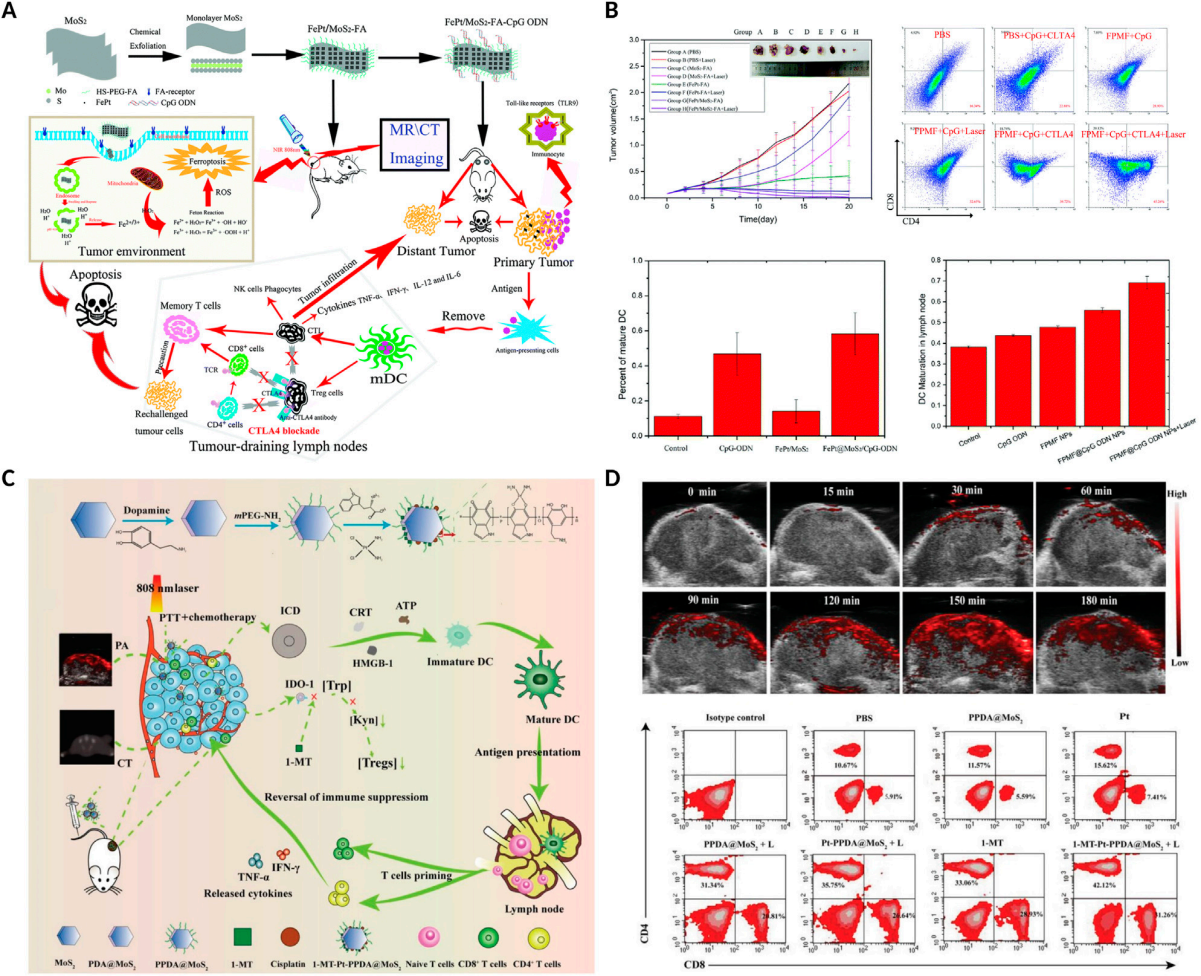
MoS_2_-based nanomaterials combined with PTT and anti-CTLA4 or 1-methyl-tryptophan (1-MT) enhanced the anticancer ability. **(A)** Schematic illustration of the FPMF@CpG ODN nanocomposites combined with chemotherapy, 808 nm laser irradiation and anti-CTLA4 *in vivo*. **(B)** The volume of tumors, the percentage of CD4^+^ and CD8^+^ T cells in tumor, and the percentage of maturation of DCs in lymph nodes after different treatments. Reproduced with permission from Ref. (Zhang D. et al., 2019) Copyright 2019, Royal Society of Chemistry. **(C)** Schematic illustration of the 1-MT-Pt-PPDA@MoS_2_ combined with 808 nm laser irradiation and 1-MT *in vivo*. **(D)** PA images of tumors at different time points after the 1-MT-Pt-PPDA@MoS_2_ complexes injection, and flow cytometric analyses of the CD4^+^ and CD8^+^ T cells in tumor. Reproduced with permission from Ref (Hu et al., 2021). Copyright The Authors. Advanced Science published by Wiley-VCH GmbH.

In recent years, MoS_2_ has been reported and rapidly employed as a photosensitizer carrier or photothermal agent due to its excellent biocompatibility and high photothermal properties (Wang et al., 2024). For example, Zhang et al. designed multifunctional FPMF@CpG ODN NCs, which was consist of folic acid (FA), FePt, MoS_2_, oligodeoxynucleotides containing cytosine-guanine (CpG ODNs) and anti-CTLA4 antibody (Zhang D. et al., 2019). The FPMF@CpG ODN NCs exhibited higher synergistic photo-chemo-immunotherapy for cancer due to the highly effective photothermal conversion of MoS_2_ nanosheets, overproduction of ROS induced by FePt, and the increase immune cells activated by CpG ODNs and CTLA4 (Figures 8A, B). In addition, in order to monitor the tumor treatment real time, Hu et al. constructed 1-MT-Pt-PPDA@MoS_2_ complexes, with excellent multimode complementary CT/PA/thermo imaging. Furtherly, the complexes could obviously inhibit the tumor growth via the synergistic photothermo-chemotherapy and activation of T cell-mediated immunotherapy to realize complete tumor eradication (Figures 8C, D) (Hu et al., 2021).

### 4.3 WSe_2_-based nanomaterials

**FIGURE 9:**
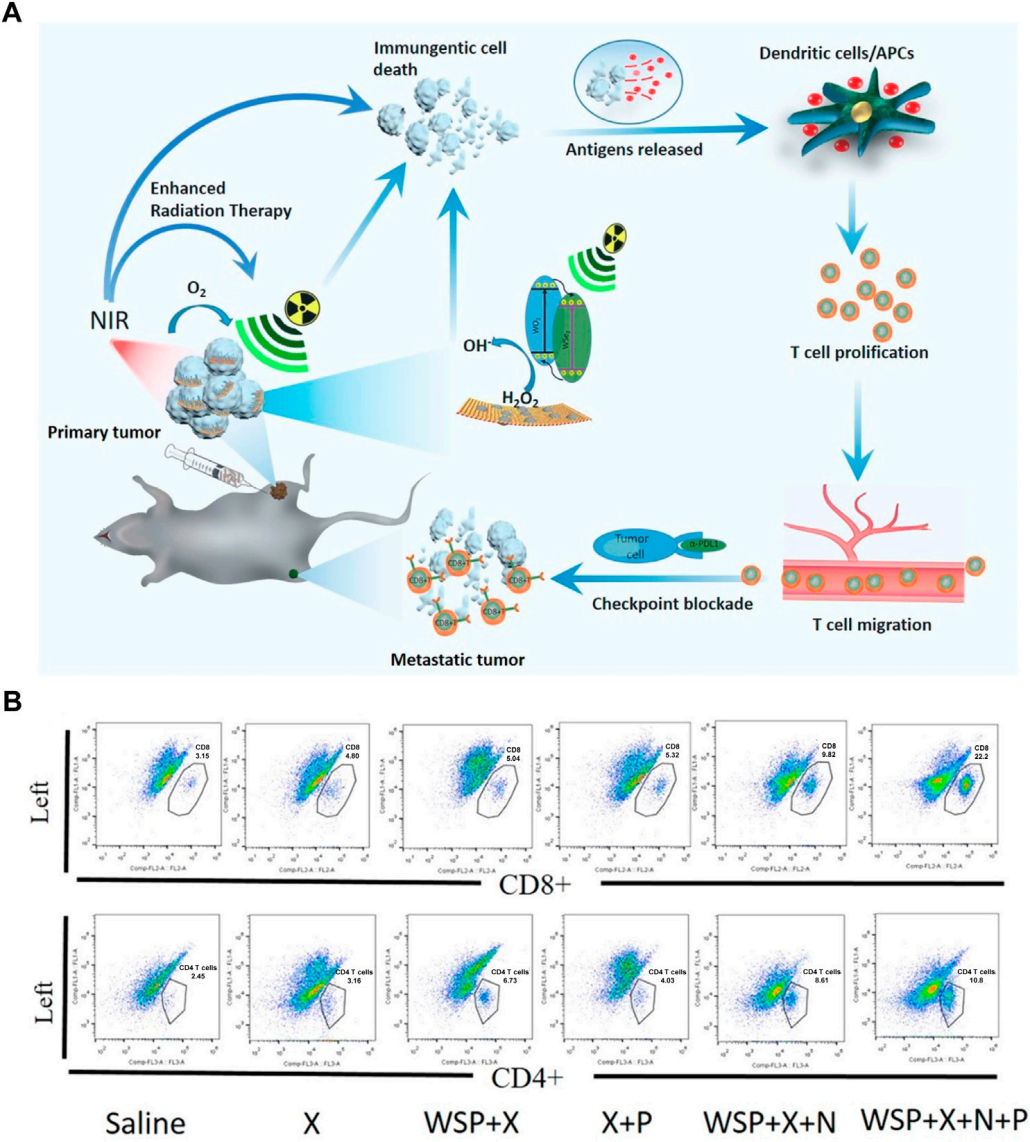
WSe_2_-based nanomaterials combined with Radiotherapy (RT), PTT and anti-PD-L1 enhanced the anticancer ability. **(A)** Schematic illustration of the WO_2.9_-WSe_2_-PEG nanoparticles combined with RT, 808 nm laser irradiation and anti-PD-L1 *in vivo*. **(B)** Flow cytometric analysis of CD8^+^ T and CD4^+^ T cells in distant tumors at the end of treatment. Reproduced with permission from Ref (Dong et al., 2020). Copyright 2020, American Chemical Society.

WSe_2_, an excellent member of the p-type TMD semiconductors and having sizable bandgap, has been widely used in building CMOS circuits (Xue et al., 2024). Furtherly, WSe_2_-based nanomaterials have exhibited high biosafety than graphene-like nanomaterials, showing enormous potential for biomedical applications (Altalbawy et al., 2025). Dong et al. designed WO_2.9_-WSe_2_-PEG nanoparticles, semiconductor heterojunction structure, to realize a synergistic RT/PTT/CBT for enhanced antimetastatic and anticancer effect (Dong et al., 2020). As shown in Figure 9A, the WO_2.9_-WSe_2_-PEG nanoparticles not only showed excellent ROS overproduction in highly expressed H_2_O_2_ TME under X-ray irradiation, but also could enhance RT outcome under the 808 nm laser irradiation via inducing hyperthermia. More importantly, the CD8^+^ T and CD4^+^ T cells in distant tumors were significantly activated in distant tumor when WO_2.9_-WSe_2_-PEG nanoparticles combined with RT/PTT and anti-PD-L1 antibody, indicating powerful immunological memory-killing and efficiently antimetastatic and anticancer effect (Figure 9B).

These results clearly illustrate that, due to their excellent physical and chemical properties, MoSe_2_/MoS_2_/WSe_2_-based nanomaterials showed high capacity to activate the CD8^+^ T and CD4^+^ T cells, thereby achieving an effective antitumor effect when combining PTT with ICT. However, the mechanisms underlying the activation of immune responses and synergistic effects of PTT in antitumor treatments remain to be elucidated.

## 5 Photothermal agents impact the immune response

### 5.1 Photothermal temperature

Evaluating the superior performance of a photothermal agent usually focuses on its photothermal stability and photothermal conversion efficiency. Cancer cells release heat shock proteins (HSP) under heat stress, and the expression of HSP facilitates the repair of cells that have been damaged by heat, contributing to the development of heat resistance in tumor cells (Jin et al., 2018). Consequently, the aforementioned property of heat resistance in tumor cells compromises the efficacy of thermal ablation at relatively low temperatures. (Toraya-Brown and Fiering, 2014; Tang X et al., 2018). In addition, an appropriate increase in temperature can increase the permeability of the tumor vasculature, thereby promoting immune cells transport to the tumor and changing the visibility of the tumor to immune cells. Therefore, the heat dose (a function of temperature and time of exposure to that temperature) has an important impact on obtaining the desired level of immune stimulation (Nomura et al., 2020). However, the exact amount of heat required to stimulate the immune system is still being explored, while small changes in heat dose may lead to gaps in the efficacy of immune stimulation (Moy and Tunnell, 2017).

**FIGURE 10:**
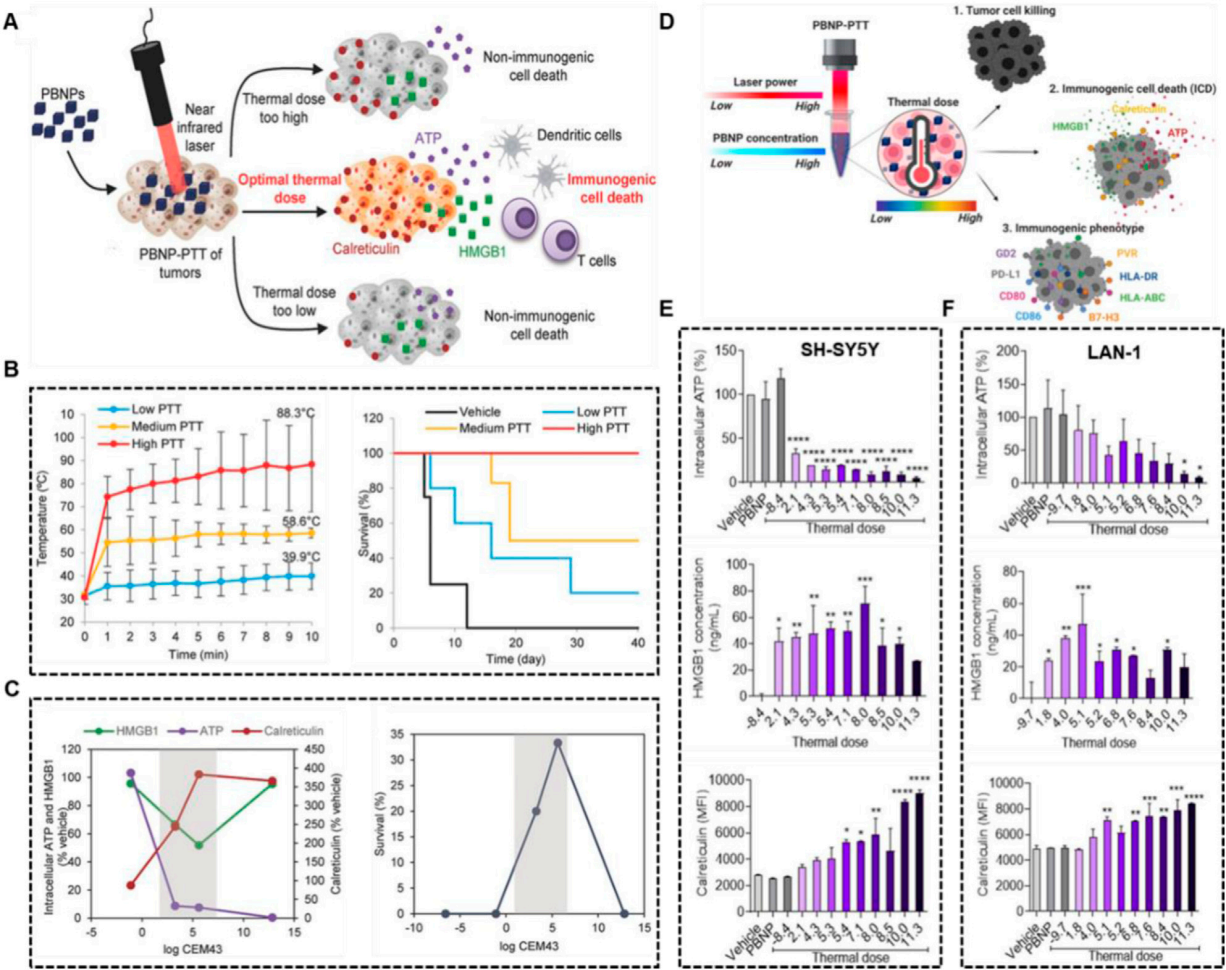
Optimal thermal window of ICD generated by PBNP-based PTT. **(A)** Schematic illustration of middle thermal dose increasing the ICD. **(B)** The tumor temperatures and survival of Neuro2a tumor-bearing mice treated with low, medium and high thermal dose PBNP-PTT. **(C)** Thermal dose [3.3–5.6, log (CEM43)] improved long-term survival of Neuro2a tumor-bearing mice. Reproduced with permission from Ref (Sweeney et al., 2018). Copyright 2018 WILEY-VCH Verlag GmbH and Co. KGaA, Weinheim. **(D)** Schematic illustration of middle thermal dose, determined by laser power and PBNP concentration, enhanced tumor cell killing and ICD. PBNP-PTT generates a thermal dose window of ICD in SH-SY5Y **(E)** and LAN-1 **(F)** cell. Reproduced with permission from Ref (Sekhri et al., 2022). Copyright 2022 by the authors.

Sweeney et al. explored the optimal temperature window for PTT-induced ICD with an animal model of neuroblastoma and demonstrated that too high and too low heat doses are not more favorable for ICD activation (Figure 10A) (Sweeney et al., 2018). Prussian blue nano-particles (PBNPs) was used as the photothermal agent, and three different heat dose groups were set up (low, <40°C; medium, <60°C and high, ≈90°C). Although higher PTT was more effective in tumor inhibition suggested by slower tumor growth and highest survival, thermal dose (3.3–5.6, log (CEM43)) initiated the innate immune responses against tumor challenge (Figures 10B, C). In addition, Sekhri et al., found that thermal doses >5 log (∑CEM43) could efficient induced ICD in SH-SY5Y cells, and thermal doses≥10 log (∑CEM43) in LAN-1 cells, which was consistent with the result that compared with LAN-1 cells, at equivalent thermal doses, cytotoxicity T cells exhibited significantly higher cytotoxicity toward SH-SY5Y cells (Figures 10D–F) (Sekhri et al., 2022).

### 5.2 Size of photothermal agents

It is well established that the size of nanoparticles influences their accumulation and retention time *in vivo*, as well as their diffusion within tumor tissues. Studies indicate that nanoparticles smaller than 5 nm are quickly cleared by the kidneys, while those larger than 100 nm may be removed by the mononuclear phagocytic cell system (Blanco et al., 2015). The size of nanomaterials used in combination PTT and ICT can influence the immune response, including cellular uptake of nanomaterials, the maturation of antigen-presenting cells (APCs), and the balance between Th2 and Th1 immune responses. Nanoparticles that are similar in size to pathogens are more readily identified and effectively internalized by APCs to trigger immune responses. Dendritic cells (DCs) preferentially internalize particles in the range of 20–200 nm, while macrophages tend to internalize larger particles (0.5–5 μm) (Zhao et al., 2014). Additionally, the photothermal conversion efficiency of nanomaterials must be considered. For instance, in the case of plasmonic nanoparticles with LSPR, the absorption, scattering, and extinction coefficients are all size- and shape-dependent (Jain et al., 2006; Kim et al., 2019). The optimal size of nanoparticles for maximizing the efficacy of PTT and ICT varies and depends on specific conditions.

The above studies suggest that different metal-based photothermal agents and their sizes may have the most suitable thermal doses for efficiently addressing the issue of cancer cells escaping immune recognition when combined PTT with and ICT.

## 6 Conclusion and future perspective

This review outlines recent developments in metal-based photothermal therapy in conjunction with immune checkpoint therapy. Noble metal-based and 2D TMDCs-based photothermal agents showed high capacity to activate the CD8^+^ T and CD4^+^ T cells to realize complete tumor inhibition and inhibit cancer cells metastasis when combined PTT with ICT. Nevertheless, metal-based photothermal therapy encounters several challenges that hinder its potential synergistic application with ICT and clinical translation, which are as follows: 1) Most metal-based photothermal agents were low photothermal conversion efficiency and biocompatibility; 2) Tumors *in situ* were little used to evaluate synergistic effects *in vivo*; 3) Noble metal-based photothermal agents and ICBs are expensive; 4) The tumor thermotolerance triggered by photothermal therapy has been established; 5) Even with NIR-II light, the irradiation sources used for most metal-based photothermal agents often struggle to penetrate the deep-seated solid tumors; 6) Current research has been limited to preclinical studies in mice and has yet to be corroborated by large-scale clinical trials (Sun et al., 2024).

As mentioned above, although the combination of PTT and ICT effectively addresses the limitations of monotherapy and brings breakthrough in cancer treatment, the development of a photothermal agent with low toxicity, high photothermal effect and cost-effective remains a bottleneck in its clinical translation. Moreover, the mechanisms underlying the activation of immune responses and synergistic effects of PTT in antitumor treatments remain to be elucidated.

Overall, the development of safe and efficient metal-based photothermal agents for enhanced synergistic ICBs therapy in cancer treatment presents both challenges and opportunities.
